# Dynamic facial emotion recognition and affective prosody recognition are associated in patients with temporal lobe epilepsy

**DOI:** 10.1038/s41598-024-53401-9

**Published:** 2024-02-16

**Authors:** Birgitta Metternich, Nina Gehrer, Kathrin Wagner, Maximilian J. Geiger, Elisa Schütz, Britta Seifer, Andreas Schulze-Bonhage, Michael Schönenberg

**Affiliations:** 1 Epilepsy Center, Department of Neurosurgery, Medical Center, University of Freiburg, Breisacher Str. 64, 79106 Freiburg, Germany; 2 Department of Psychiatry and Psychotherapy, University Hospital Tübingen, Tübingen, Germany

**Keywords:** Epilepsy, Human behaviour

## Abstract

Deficits in facial emotion recognition have frequently been established in temporal lobe epilepsy (TLE). However, static, rather than dynamic emotion recognition paradigms have been applied. Affective prosody has been insufficiently studied in TLE, and there is a lack of studies investigating associations between auditory and visual emotion recognition. We wished to investigate potential deficits in a dynamic morph task of facial emotion recognition and in an affective prosody recognition task, as well as associations between both tasks. 25 patients with TLE and 24 healthy controls (CG) performed a morph task with faces continuously changing in their emotional intensity. They had to press a button, as soon as they were able to recognize the emotion expressed, and label it accordingly. In the auditory task, subjects listened to neutral sentences spoken in varying emotional tones, and labeled the emotions. Correlation analyses were conducted across both tasks. TLE patients showed significantly reduced prosody recognition compared to CG, and in the morph task, there was a statistical trend towards significantly reduced performance for TLE. Recognition rates in both tasks were significantly associated. TLE patients show deficits in affective prosody recognition, and they may also be impaired in a morph task with dynamically changing facial expressions. Impairments in basic social-cognitive tasks in TLE seem to be modality-independent.

## Introduction

Temporal lobe epilepsy (TLE) is the most common type of focal epilepsy, comprising 2/3 of all focal epilepsies^1^. People with (focal) epilepsy (PW(F)E) suffer from neuropsychological deficits^2^. Intact cognitive abilities are highly relevant for everyday functioning and impairments can significantly reduce quality of life^3–8^. Deficits in social cognition may worsen social integration^9^, but are under-researched in PWFE to date.

### Basic social cognition and the temporal lobes

The term “social cognition” subsumes perceptual and mental processes that are essential for successful social interactions, e.g., basic visual and auditory emotion recognition. The temporal lobes are important for basic visual and auditory emotion recognition^10–12^. Visual emotion recognition usually involves inferring emotional states from facial expressions, taking into account relevant facial regions, such as the eyes and the mouth. Mesiotemporal brain areas may be relevant to accurate facial emotion recognition^13^. The amygdala is a mesiotemporal structure which plays an important role in (facial) emotion processing^14^. There is evidence for the amygdala being involved if inferences to the emotional state of a person are to be derived from the eye region^15^. Neurological patients with uni- and especially bilateral amygdalar damage show more impaired facial emotion recognition, in particular where fear and other negative emotions are concerned, than patients with other kinds of brain damage or healthy subjects^10,15,16^. Accurate recognition of emotional signals is not only relevant concerning facial expressions, but also plays an essential role in verbal interactions where the auditory domain is important. The ability to understand spoken utterances and interpret their meaning, does not only rely on the syntactic structure of sentences or the semantic meaning of words, but also on the prosodic content of an utterance^11^. Prosody comprises modulation of speech through pitch, volume, tone and intonation, through which the semantic and affective content of speech can be inferred. The understanding of prosody seems to rely on an extensive neural network which comprises the posterior temporal lobe as well as subcortical structures^11,12^. Functional magnetic resonance imaging and transcranial magnet stimulation studies have provided evidence for the importance of the right hemisphere for processing affective prosody^17–19^. Right posterior lateral temporal lobe lesions may be associated with impairments in the comprehension of emotional prosody^20^. However, other studies cast doubt on the right-hemispheric lateralization of emotional prosody processing and point to the involvement of a complex network of left- and right-hemispheric areas in such processes^21^.

### Basic emotion recognition in TLE

In TLE patients, seizure activity and—depending on individual etiology—structural lesions of the cortex can impair functioning of temporo-lateral as well as mesiotemporal areas including the amygdalae. Hence, deficits in basic facial emotion recognition have been frequently documented^22^, and evidence from eye-tracking studies suggests that during tasks of facial emotion recognition patients with TLE show abnormal eye-movement patterns, which may be associated with emotion recognition performance^23^. In such investigations, participants are usually presented with faces showing one of the six basic emotions. TLE patients tend to perform worse than healthy control subjects on this task^24,25^. Literature reviews suggest a more pronounced deficit for negative emotions in patients with TLE, perhaps especially for fear^10,22^. However, methodical issues in a number of studies render the assumption of such a specific deficit questionable. In the vast majority of prior research, static paradigms of facial emotion recognition have been applied. Using novel morph-tasks with slowly changing faces, from neutral to full emotional expressions^26–29^, allows not only for comparing correct answers between PWFE and healthy controls, but also the minimal degree of emotional expression which is needed for a correct response. Considering ecological validity, this is of high relevance for everyday social interactions, where people rarely show full facial expressions of basic emotions. Instead, subtle signals have to be decoded and interpreted correctly within a limited time frame.

For basic auditory emotion recognition, affective prosody recognition tasks can be applied. Affective prosody recognition can be assessed in a standardized manner by listening to spoken sentences that are semantically neutral. These are spoken with differing emotional prosody, and subjects are asked to identify the respective emotion^11^. Due to functional disturbances of relevant brain areas (see above), patients with TLE may also show deficits in recognizing affective prosodic content of acoustic stimuli ^10,17^. However, a very limited number of studies have tested affective prosody recognition with a standardized paradigm as described above, and included more than ten adult epilepsy patients^11,19,30–35^. Only some of these studies showed a deficit in prosodic emotion recognition in the included epilepsy patients. Whether a specific deficit for the recognition of fear or all negative emotions from prosody exists in patients with TLE, is questionable. For here, the same methodical concerns apply as in the studies on facial emotion recognition.

Even fewer studies have compared performance across the auditory and visual domain^10,17^. Three of these found cross-modal deficits^30,31,33^. Only one study including preoperative patients conducted a correlation analysis and reported a significant association between emotional prosody recognition and facial emotion recognition^31^. Hence, more research into cross-modal impairment of emotion recognition in patients with focal epilepsy is needed. So far, only tasks of prosody recognition of individual emotions have been applied in focal epilepsy research, but no tasks of prosody *discrimination*, which is a more basic process than emotion labelling.

The present study investigated facial emotion recognition in TLE patients with an animated morph task. Prosodic emotion recognition was also tested with a standardized paradigm, and we aimed to test the following hypotheses:

1. Patients with TLE show a deficit in prosodic emotion recognition compared to healthy control subjects. The more basic process of affective prosody discrimination is not expected to differ between groups.
2. Based on the literature on other patient groups suffering from structural and functional mesiotemporal abnormalities, it is expected that TLE patients also show a deficit in a dynamic facial emotion recognition task compared to healthy control subjects, i.e., average combined accuracy and speed indices are lower than those of healthy control subjects.
3. Emotion recognition performance in TLE is expected to be cross-modal, i.e., performances in the auditory and visual domain are expected to be statistically related.

## Methods

### Subjects

Twenty-five patients with active TLE from the Epilepsy Center of Freiburg University Medical Center took part in the study. Parts of the sample were recruited from a cross-section study on social cognition in focal epilepsy^36^. Patients underwent interdisciplinary presurgical assessment. Diagnosis of TLE was based on data from video-EEG-monitoring, as well as MRI. Twenty-four healthy control subjects were recruited via advertisements on the Medical Center’s intranet pages. Exclusion criteria were: (i) severe psychiatric disorders, (ii) neurodegenerative disorders, (iii) other types of epilepsy, (iv) age below 18 years, (v) insufficient grasp of the German language, (vi) verbal intelligence: IQ ≥ 85.

One patient was excluded due to severe depression, three patients due to idiopathic generalized epilepsy or an extra-temporal epilepsy focus. One patient did not complete the animated morph task, but was not excluded from the study, as he completed all other assessments. Three patients had not experienced any seizures during the previous two months, but had experienced regular seizures beforehand. One patient did not receive anticonvulsive medication at the time of their neuropsychological evaluation. For reasons of matching, the two control subjects with the highest verbal IQ scores had to be excluded.

The study was approved by the Ethics Committee of Albert-Ludwigs-University Freiburg (No. 206/17) and conducted in accordance with the Declaration of Helsinki. All patients provided informed written consent before participation.

### Instruments

#### Mehrfachwahl-Wortschatz-Intelligenztest (MWT-B)

Verbal-crystallized intelligence was estimated with a vocabulary test, the Mehrfachwahl-Wortschatz-Intelligenztest (MWT-B)^37^. Subjects are asked to recognize and mark each genuine German word among four distractors.

#### Beck Depression-Inventory, 2nd edition (BDI-II)

Depressive symptoms were assessed with the German version of the Beck Depression Inventory, 2nd edition^38^.

#### Affective prosody recognition

The prosody task was adapted from Nazarov and colleagues^39^.

Semantically neutral spoken sentences were presented in either an emotional (fearful, happy, sad, angry, disgusted) or neutral tone. Stimuli originate from the “Berlin Database of Emotional Speech.”^40^. In the recognition task, subjects had to choose which emotion was expressed out of a choice of six (see above). There was no time limit on this task. The recognition task included three practice trials and 72 experimental trials (12 repetitions of each emotion, 10 speakers). In a further task (emotion discrimination), the same sentence was spoken by two different persons, and the task was to state whether the emotional expressions are the same or different. The discrimination task included two practice trials and 20 experimental trials (10 trials with the same and 10 with different emotions, two different speakers on each trial). This task taps a more basic level of perception than *identifying* an emotion correctly from prosody.

#### Animated morph task

Facial emotion recognition was assessed with an animated morphing paradigm. The task consists of a series of computerized movies, depicting facial expressions changing successively from neutral (0%) to one of four emotional expressions (100%) in 2% increment steps. Digitized color photographs depicting four affective states (angry, happy, fearful, sad), selected from the Radboud Faces database (University of Nijmegen^41^), served as stimulus material for the experimental trials. Emotional expressions are altered using a morphing procedure (FantaMorph software, Abrosoft, Beijing, China). Every image is presented for 200 ms. The experiment consists of 40 emotion sequences (10 models (5 male/5 female) × 4 emotions) and takes about 20 min. to complete. Participants were asked to press a button as soon as they were able to detect an emotional expression. The sequence was then immediately stopped, the face disappeared, and the perceptual intensity as well as the selected emotion category were recorded. This task has been applied in previous studies of the work group^26,28,42^.

In order to assess performance on the morph task, the balanced integration score (BIS^43^) was applied, a composite performance score including accuracy of emotion identification as well as the speed of identification (expressed here as the grade of intensity needed for a correct identification). The BIS is the difference in standardized mean correct reaction times and the proportion of correct responses^43^.

### Statistical analyses

For the main analyses, a one-way ANOVA was calculated for the morph task. For the prosody task and for secondary analyses, RM-ANOVAs were computed across prosody subtasks and the different emotions in both prosody recognition and the morph task, in order to explore whether potential deficits in the TLE group are diffuse or specific to individual emotions, i.e., whether an interaction between subject group and type of emotion appeared. The included variables were for (a) the prosody task: group status as a fixed factor and the two task types (prosody recognition and prosody discrimination) on the repeated measures factor, (b) the prosody recognition task: group status as a fixed factor and the five emotions (anger, fear, sadness, joy, disgust) plus the neutral expression on the repeated measures factor, and c) the morph task: group status as a fixed factor and the four emotions (anger, fear, sadness, joy) on the repeated measures factor. If applicable, post-hoc t-tests were computed.

Pearson correlations were computed in order to test for an association between auditory and visual emotion recognition accuracy, as well as the defined daily dose (DDD)^44^ of anticonvulsant medication and the measures of social cognition. Spearman’s rank correlations were computed in order to test for an association between depression scores (BDI-II) and auditory as well as visual emotion recognition.

### Ethics approval

The study was approved by the Ethics Committee of Albert-Ludwigs-University Freiburg (No. 206/17).

### Consent to participate

All participants gave informed written consent.

## Results

### Demographic and clinical variables

Table 1 shows clinical and demographic characteristics of TLE patients and demographic parameters for healthy control subjects. No significant group difference emerged between TLE patients and healthy controls for gender, age and verbal IQ (see Table 1). TLE patients as a group displayed significantly more depressive symptoms than the control group. More patients than healthy control subjects were currently unemployed or retired. Relationship status did not differ significantly between groups.

**Table 1:** Demographic and clinical features of TLE and CG.

Variable	TLE *(n* = *25)*		CG *(n = 24)*		
	*M*	*SD*	*M*	*SD*	*p*
Age	35.8	14.2	32.6	8.6	0.35
Verbal intelligence (IQ)	106.1	14.3	111.3	12.2	0.18
Symptoms of depression (BDI-II)	10.24	7.4	4.0	2.9	0.00
Epilepsy duration	14.8	11.5			
	n		n		
Gender	13 female/12 male		16 female/8 male		0.30
Currently in a relationship	17 yes/8 no		14 yes/10 no		0.56
Employment status (employed or in training)	20 yes/5 no		24 yes/0 no		0.02
Employed	15		15		
Trainee/student	5		9		
Unemployed	2		0		
Retired	3		0		
Seizure type^1,2^					
No seizures	3				
Focal aware seizures	9				
Focal unaware seizures	19				
Bilateral tonic–clonic seizures	7				
Focus lateralization					
Dominant hemisphere	13				
Non-dominant hemisphere	10				
Bilateral	1				
Inconclusive	1				
MRI pathology^1^					
Unspecific (WML, gliosis, signal alterations)	6				
Limbic encephalitis	2				
Tumor/cavernoma	8				
Focal cortical dysplasia	3				
Amygdalar hyper-/hypoplasia	6				
Hippocampal sclerosis/hyperintensity	2				
Temporal polar atrophy	1				
Parenchymal defect	4				
None	2				
Anticonvulsive medication count					
Monotherapy	13				
Polytherapy	11				
No medication	1				

*BDI-II* Beck Depression Inventory (revised 2nd version), *CG* Control group, *TLE* temporal lobe epilepsy, *WML* white matter lesions.

^1^Multiple classification possible.

^2^Seizures during the previous 2 months.

### Facial emotion recognition (morph task)

The ANOVA showed a statistical trend towards a significant effect of group, i.e., in tendency, patients’ BIS, combined accuracy and speed, was lower than the control group’s mean BIS (see Table 2). Secondary RM-ANOVAs on accuracy scores revealed no significant interaction between group and type of emotion (F = 0.42, p = 0.65).

**Table 2:** Mean BIS for morph task according to group status.

Morph task	Group	N	Mean z-score (SD)	Statistics *p*
BIS	TLE	24	− 0.34 (1.52)	0.08
	CG	24	0.34 (1.03)	

*BIS* balance integration score (difference in z-scores for speed and accuracy across all conditions), *CG* healthy control group, *TLE* patients with temporal lobe epilepsy.

### Emotion recognition from prosody (prosody task)

**Figure 1:**
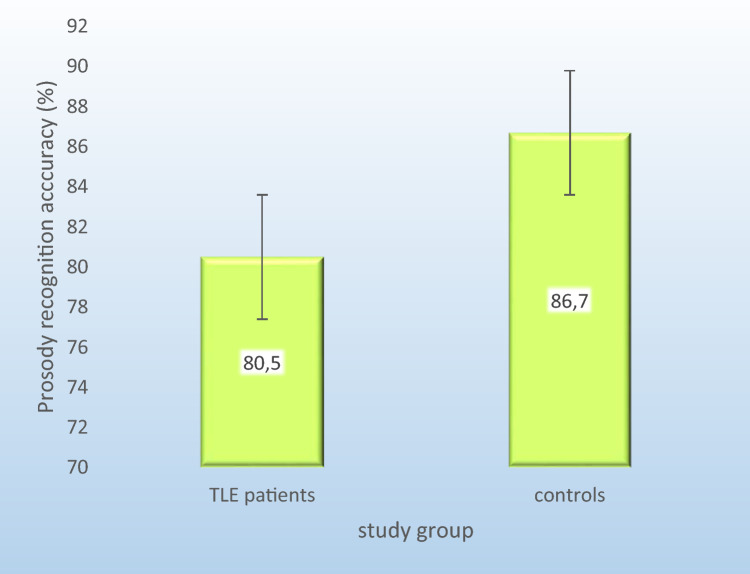
Percentage of correctly identified emotions out of all emotions presented in the prosody task divided by subject group. *CG* Healthy control group, *TLE* Patients with temporal lobe epilepsy. Mean difference significant at p < 0.05.

The RM-ANOVA revealed a significant effect of prosody task type (F = 67.7, p < 0.001), i.e., performance on prosody discrimination was superior to prosody recognition performance across groups. Furthermore, a significant interaction effect group x task emerged (F = 4.7, p = 0.04). Post-hoc t-Tests showed a significant difference between TLE patients and controls with the former performing significantly worse on prosody recognition (t = − 2.3, p = 0.03), i.e., on average, TLE patients were less accurate in identifying emotions from prosody than healthy controls (see Fig. 1), but not on prosody discrimination (t = − 0.2, p = 0.85; see Fig. S9 for an illustration of the interaction).

Secondary RM-ANOVAs revealed no significant interaction between group and type of emotion (F = 0.58, p = 0.67) for recognition accuracy.

### Correlation analyses

**Figure 2:**
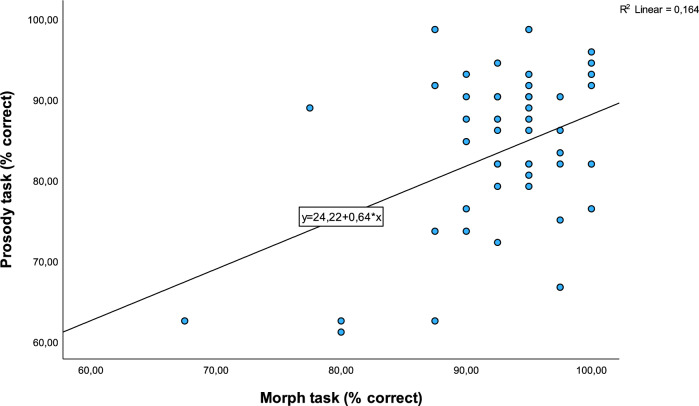
Scatter plot for the correlation between the percentages of correct answers in the morph task and the prosody task.

The Pearson correlation coefficient for accuracy scores in affective prosody recognition and the morph task was significant (r = 0.41, p = 0.04, see Fig. 2). There were no significant associations between depression scores (BDI-II) or verbal IQ and either morph or prosody task performance (Spearman’s rank correlations for BDI-II and Pearson correlations for IQ), all coefficients r < 0.2, all p-values > 0.2). No significant correlations emerged for either the morph task or affective prosody recognition and drug-load (DDD) (Pearson correlations, all coefficients r < 0.2, all p-values > 0.4).

### Additional analyses

In order to investigate whether lateralization had an effect on prosody and morph task performance, we compared patients with TLE in the dominant (N = 13) and in the non-dominant hemisphere (N = 10) on both tasks. No significant between-group differences were detected either for the prosody task (d = − 0.04, p = 0.92) or for the morph task (d = 0.69, p = 0.12).

## Discussion

The present investigation shows a deficit in affective prosody recognition in patients with active TLE compared to a group of healthy control subjects. This finding is consistent with prior research showing deficits in prosodic emotion recognition in patients with TLE^11,31,33^ or after temporal lobectomy^11,30,32^, and adds to the modest number of studies investigating this aspect of emotion recognition in patients with TLE. Not all previous research has found impairment in recognizing emotional prosody in PWFE^19,34,35^. These negative results could be due to small subgroup sizes and differences in sampling, e.g., Adolphs and colleagues^34^ compared their patients to brain-damaged controls. Studies with larger samples or studies that included a comparison between their entire patient sample with a group of healthy control participants seem to have been more successful in discovering significant group differences favoring healthy controls^11,30,31,33^. TLE patients in the present study were also less accurate in identifying emotions from prosody than healthy control subjects. However, TLE patients did not differ from healthy controls regarding *discrimination* of emotional prosody. The RM-ANOVA revealed a significant group × task type interaction. Prior research in TLE has focused on active identification of emotions from prosody. Discrimination tasks have not been included, to our knowledge. Therefore, the present study has shown a dissociation between a comparison of prosodic information (prosody discrimination) and emotion labelling from prosody (emotional prosody recognition) in adult patients with TLE for the first time. This is interesting in so far as it shows that TLE patients’ impairment does not include the more basic process of prosodic discrimination, but merely the higher process of labelling the emotions expressed by prosodic content.

The present results show a trend towards reduced performance on a dynamic facial emotion recognition task in patients with TLE. Post-hoc power analyses showed that statistical power may have been too low to detect the medium-sized effect between the TLE and the control group (see “Limitations”). Previous research has focused on static, rather than dynamic paradigms of facial emotion recognition^10,22^. Deficits in static facial emotion recognition have been established in various studies including patients with TLE^10,22,23^. About half of the 31 studies included in Bora and Meletti’s systematic review^22^ only reported on postsurgical patients after temporal lobectomy. However, TLE patients were impaired regarding emotion recognition regardless of whether or not they had received surgery. Very few investigations have included dynamic tasks of facial emotion recognition: Tanaka and colleagues^45^ used a video-based task, in which the actors displayed an emotional expression from neutral for 2 s. and then returned to a neutral expression. The authors found a deficit in emotion recognition in the included patients with TLE and a further group with temporal lobectomy. A similar task was applied in a study by Young and colleagues^46^, producing similar results. Sedda and colleagues applied a task including morphed faces of varying degrees of emotional expression^47^. However, only one static face was shown at a time, and subjects had to label the shown emotion. Ammerlaan and colleagues^48^ used a task similar to the one in the present study, where faces displaying emotions gradually changed in emotional intensity. However, unlike the animated morph task in the present study, in the paradigm used by Ammerlaan and colleagues, no reaction times were measured, i.e., participants could simply watch the video until the final degree of intensity was reached and shown and then proceed with the task of labelling the displayed emotion. In contrast to this, during the morph task used in the present study, subjects were required to press a button, as soon as they believed they had recognized the displayed emotion. To our knowledge, the latter kind of animated morph task has not been applied in epilepsy research so far. The investigations cited above and the present results suggest a possible deficit for dynamic facial emotion recognition in TLE. TLE patients may show a deficit in a dynamic task of facial emotion recognition that combines the required intensity of facial expressions and correct emotion identifications. Such a task comes closer to everyday requirements in social cognition than a static emotion recognition task. In face-to-face interactions, we rarely see the full degree of an emotional expression in the person with whom we are interacting. Instead, we have to decode subtle emotional signals within a limited time frame and respond in an adequate manner. It will be important to investigate TLE patients’ deficits in such dynamic tasks more closely in the future.

Emotion recognition impairment discovered on the prosody task as well as the trend towards impaired performance on the morph task, was not confined to specific emotions. Instead, it can be hypothesized that patients show a more diffuse impairment across all emotions. Despite many previous studies having discovered that PWFE are impaired in recognizing fear, for example, there is no convincing evidence to suggest a specific deficit in fear recognition in this patient group^22^. Firstly, most studies have found deficits in recognizing other emotions as well^10,22^, secondly, repeated-measures analyses of variances have not routinely been conducted. Many studies seem to have conducted individual between-group comparisons for each emotion (with or without subsequent Bonferroni corrections) or a MANOVA (e.g.,^30,31,33,49,50^). This is problematic in so far, as only a repeated-measures analysis of variance allows for the detection of a significant interaction effect group × emotion, which is the precondition for subsequent individual comparisons^51^. If such an interaction effect cannot be shown, a pervasive deficit in TLE patients that is independent of the type of emotion seems more plausible.

Although this was not the main focus of the present study, we conducted secondary analyses, which showed the absence of an interaction effect between subject group and type of emotion concerning both, the visual and the auditory task. The lack of such an interaction effect points at an impairment across various emotions, rather than a specific deficit, but this notion needs to be explored further, as the lack of an interaction effect alone cannot be considered as evidence for a diffuse impairment. Therefore, it has to be concluded that we found no evidence for a specific deficit in emotion recognition in TLE in our sample. Bora and Meletti^22^ came to the following conclusion in their systematic review: As in comparison with healthy controls, TLE patients were significantly impaired on labelling fear, disgust, sadness, anger, surprise as well as happiness (even if the effect size for happiness was small (d = 0.2)), the authors concluded that chronic TLE leads to impaired recognition of *all* basic emotions.

Auditory and visual emotion recognition performance seems to be associated, as the significant correlation between the rates of correct identifications in both tasks shows. Although it is important to know whether PWFE show cross-modal deficits in emotion recognition, this issue has not been addressed sufficiently in prior research. In Monti and Meletti’s systematic review^10^, most of the included studies investigated either facial or prosodic emotion recognition, and out of the six studies exploring both modalities in more than one adult patient, four reported correlation analyses^30,31,33,34^. Out of these, three found significant correlations between auditory and visual measures of emotion recognition ^30,31,33^. These results show that the association between emotion recognition from prosody and from faces is under-researched, but taking into account present and past results, a significant association between the two modalities seems probable.

When patients with TLE in the dominant and non-dominant hemispheres were compared in our sample, there were no significant differences regarding performance on either of the tasks, which is consistent with most prior study results. Nevertheless, the analyses were underpowered due to the small N of the subgroups (see “limitatons”). Alba-Ferrara and colleagues^17^ postulate a key role of the right hemisphere in processing affective prosody. However, concerning lateralization, results across studies are less consistent than could be assumed from the results of functional imaging investigations in healthy subjects. None of the studies found performance impairment in left-sided TLE, and at least preliminary evidence for a relative deficit in right-sided TLE emerged in some studies^33,52,53^. However, most studies did not show a significant effect of lateralization, which may also be due to insufficient numbers of subjects in subgroup comparisons^10,17^. Another explanation for inconsistent results regarding lateralization could be the existence of reorganization processes and plasticity in PWFE, possibly via recruitment of contralateral cortical areas^19^.

### Limitations

The included patient sample is of moderate size. Power may have been too low in order to detect between-group medium effect sizes on the morph task. Post-hoc power analyses revealed a power of 1 − β = 0.43. With a larger sample size, the group effect on the BIS for morphing may have become significant, rather than remaining a mere trend towards significance. Power was slightly higher for the prosody task (recognition) with 1 − β = 0.62, but low for the tiny effect on prosody discrimination (1 − β = 0.05). However, power was high for the interaction effect of the RM-ANOVA for the two prosody subtasks (1 − β = 0.99) as well as the interaction effects on the RM-ANOVAs for the individual emotions for morphing and prosody recognition (both 1 – β ≈ 0.8). The same holds true for the correlation between visual and auditory emotion recognition (1 − β = 0.85) so that these latter analyses should not have been impacted by the moderate sample size. However, analyses would have been underpowered to detect small, rather than medium correlations, such as were found in the remaining correlation analyses (r = 0.04 to 0.2). Sub-samples of patients with TLE in the dominant and non-dominant hemispheres were small. Therefore, subgroup comparisons according to lateralization were underpowered. Post-hoc power analyses revealed a power of 1 − β = 0.3 for medium effect sizes.

Our auditory and visual emotion recognition tasks were not parallel in design, i.e., only the morph task measured speed as well as accuracy, and both tasks did not include the same set of emotions. Therefore, we were unable to compare individual emotion recognition across tasks. Quasi-experimental studies are vulnerable to confounders. The participant groups included in the present study did not differ significantly in verbal IQ, age or gender. However, TLE patients displayed significantly increased symptoms of depression in comparison with the healthy control group. Nevertheless, correlation analyses showed that symptoms of depression were not significantly associated with our outcome parameters. Even though there were no significant group differences on verbal IQ, we conducted a correlation analyses between verbal IQ scores and social-cognitive parameters, which yet again did not reveal any significant associations. As anticonvulsive medication can result in adverse cognitive effects, the results of the present study could have been impacted by this. However, the lack of an association between DDD and the two measures of social cognition renders this bias less probable, although very small associations may not have been detected due to power restrictions (see above).

### Summary and outlook

The present study shows impaired affective prosody recognition performance in patients with an active TLE. Furthermore, possibly, a deficit in dynamic facial emotion recognition may exist in this patient group. Even if the present study’s results merely provided a trend towards statistical significance for this measure, favoring the control group, dynamic facial emotion recognition paradigms appear to be a fruitful and somewhat more realistic approach of everyday demands on social cognition than static paradigms. As such, they should be researched in more detail and with larger patient cohorts. Furthermore, larger subgroups split by focus localization, lateralization and amygdalar involvement should be included. Studying patients with discrete lesions could increase our understanding of temporal lobe function in social cognition in general. In order to be able to examine deficits across modalities in more detail, auditory and visual tasks should employ a parallel design. Cross-modal social cognitive deficits in TLE may predict deficits in everyday social cognition, i.e., social functioning. Hence, the association between basic emotion recognition and social functioning needs to be explored further in future studies.

### Supplementary Information

Supplementary Figure S1.

Supplementary Figure S2.

Supplementary Figure S3.

Supplementary Figure S4.

Supplementary Figure S5.

Supplementary FigureS6.

Supplementary Figure S7.

Supplementary Figure S8.

Supplementary Figure S9.

## Data Availability

The datasets used and analysed during the current study are available from the corresponding author on reasonable request.
