# Supplementary figures and images for: Dynamic facial emotion recognition and affective prosody recognition are associated in patients with temporal lobe epilepsy
Source: Sci Rep. 2024 Feb 16;14:3935. doi: 10.1038/s41598-024-53401-9 (PMC10873350; doi:10.1038/s41598-024-53401-9)

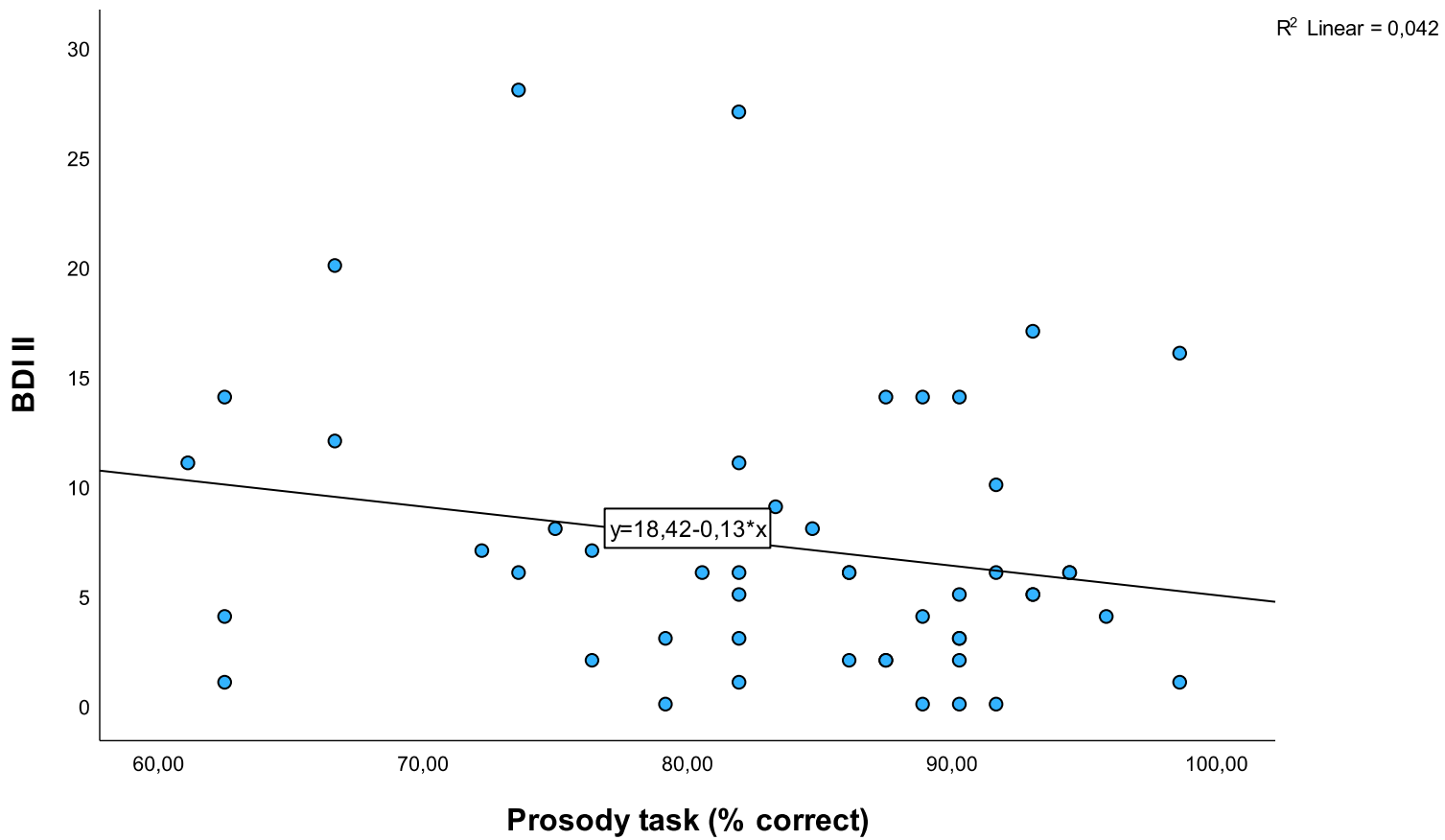

Supplement: Supplementary file 1 — Supplementary Figure S1. [file 41598_2024_53401_MOESM1_ESM.pdf]

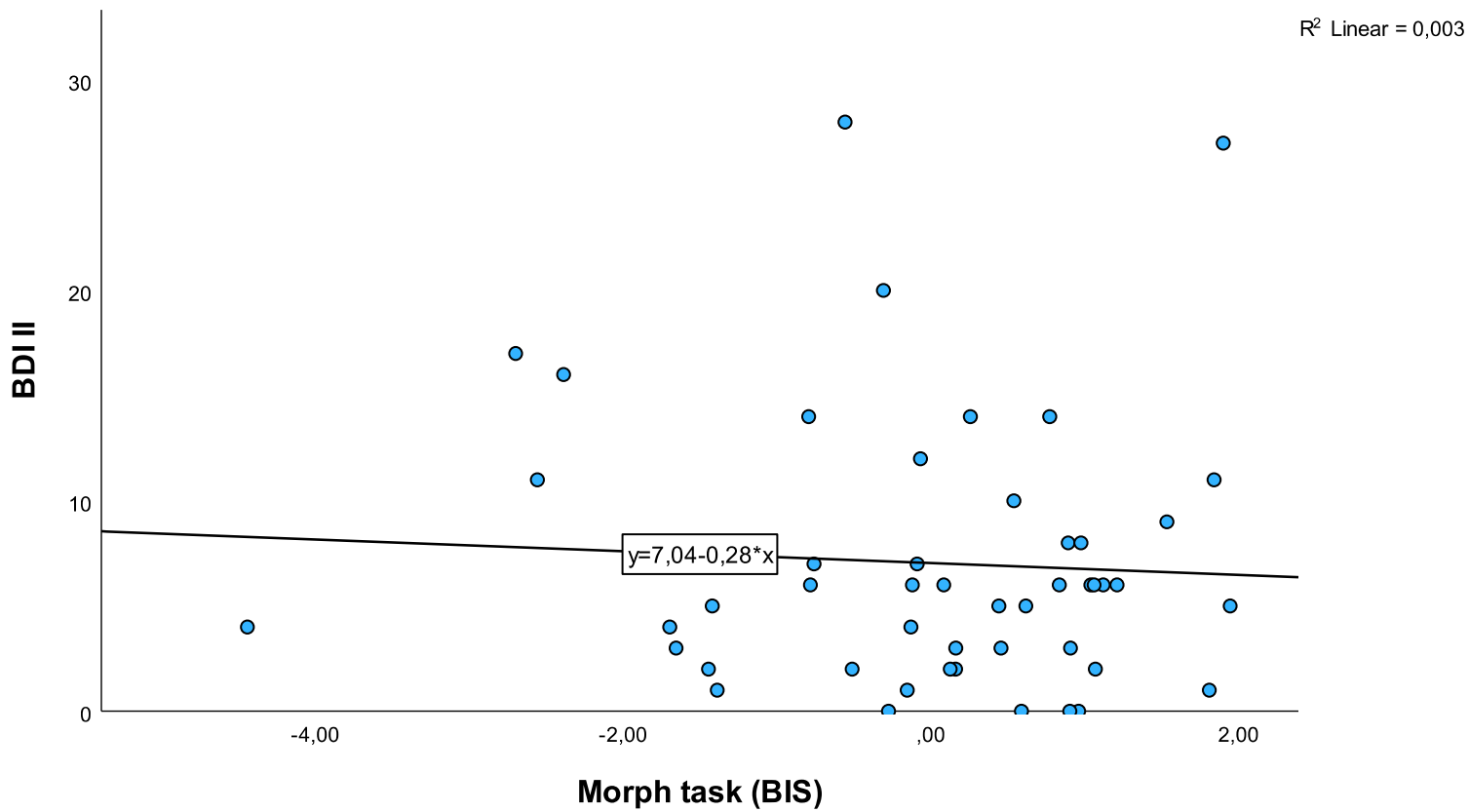

Supplement: Supplementary file 2 — Supplementary Figure S2. [file 41598_2024_53401_MOESM2_ESM.pdf]

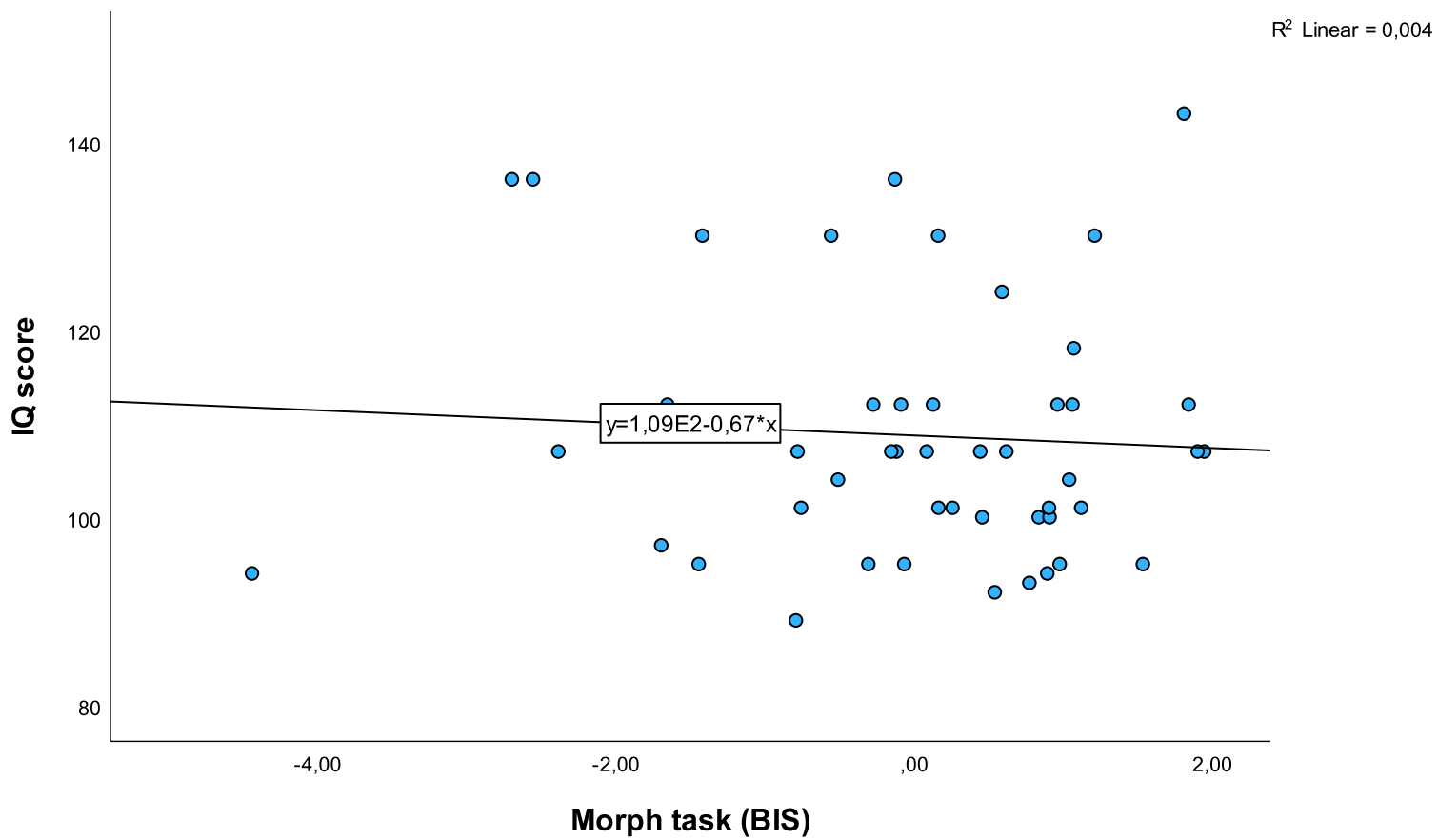

Supplement: Supplementary file 3 — Supplementary Figure S3. [file 41598_2024_53401_MOESM3_ESM.pdf]

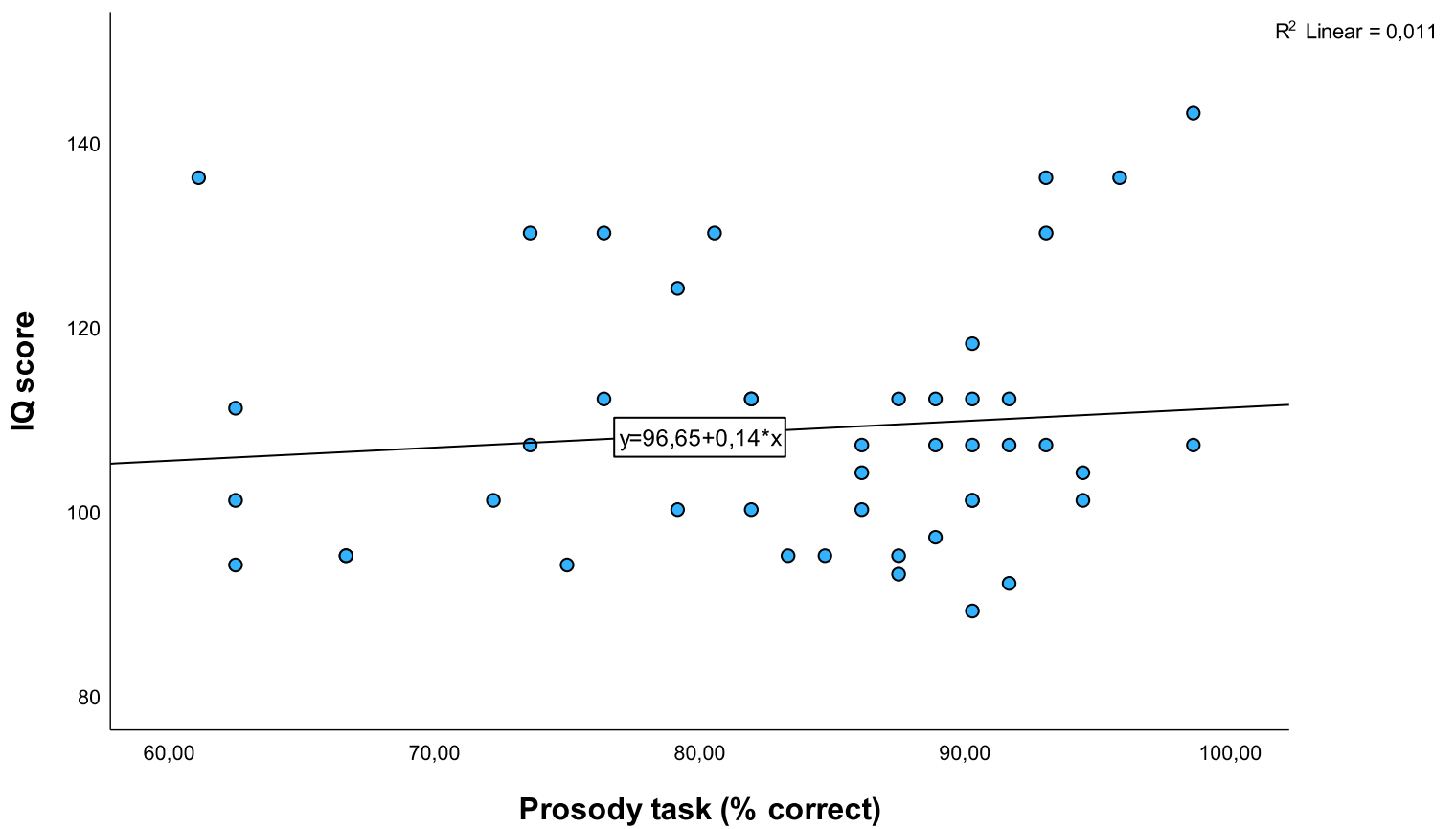

Supplement: Supplementary file 4 — Supplementary Figure S4. [file 41598_2024_53401_MOESM4_ESM.pdf]

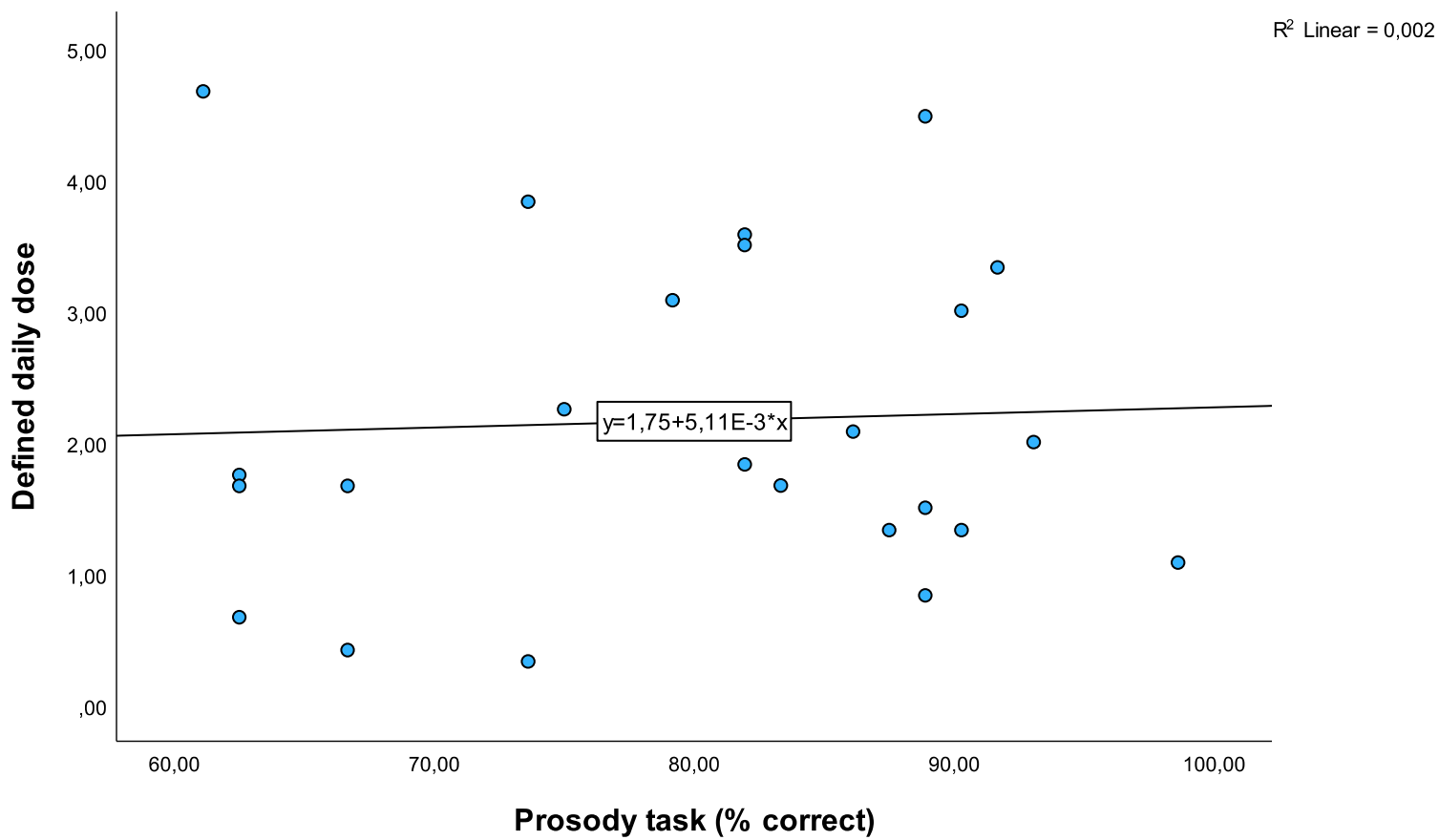

Supplement: Supplementary file 5 — Supplementary Figure S5. [file 41598_2024_53401_MOESM5_ESM.pdf]

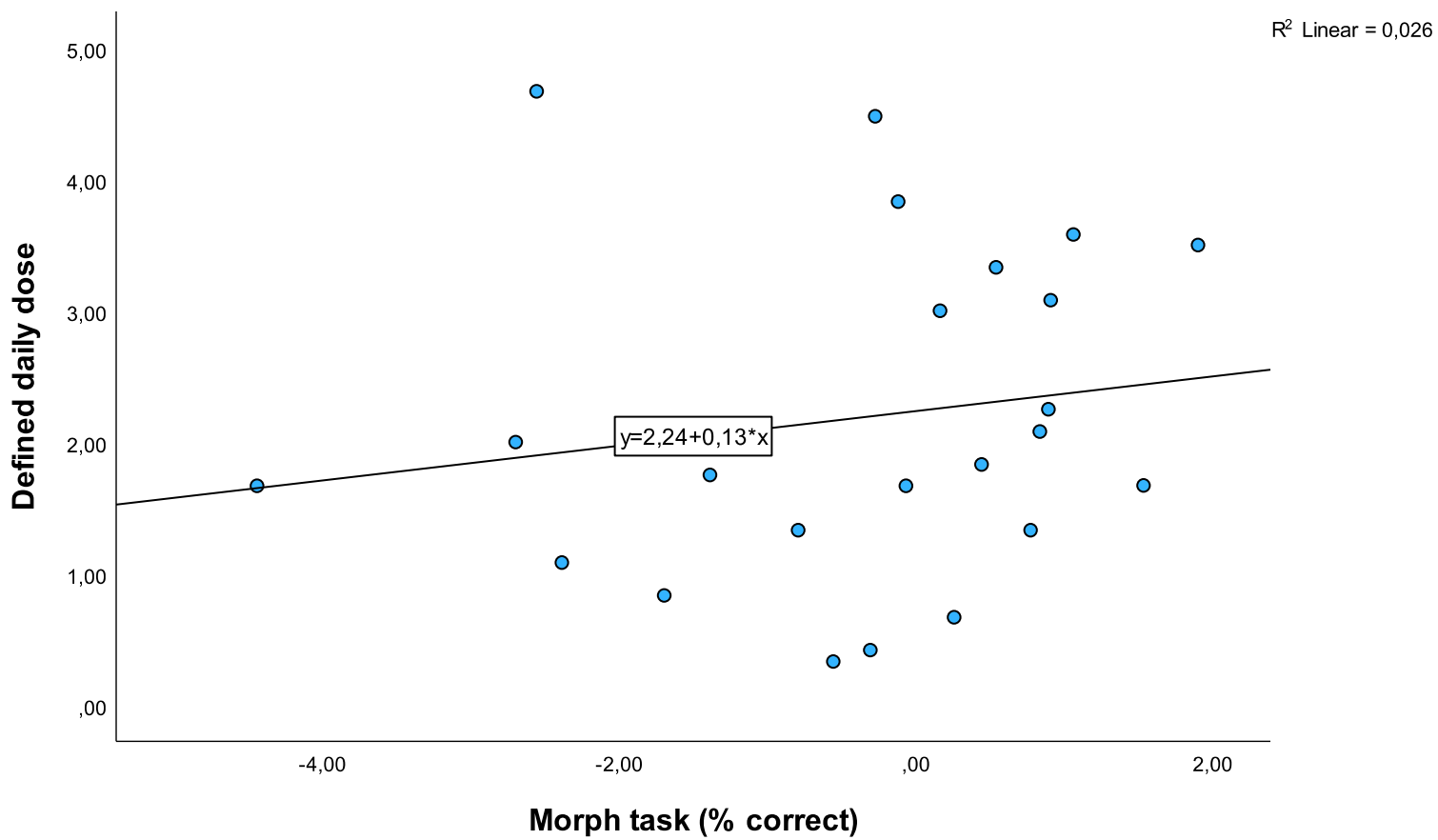

Supplement: Supplementary file 6 — Supplementary FigureS6. [file 41598_2024_53401_MOESM6_ESM.pdf]

TLE

controls

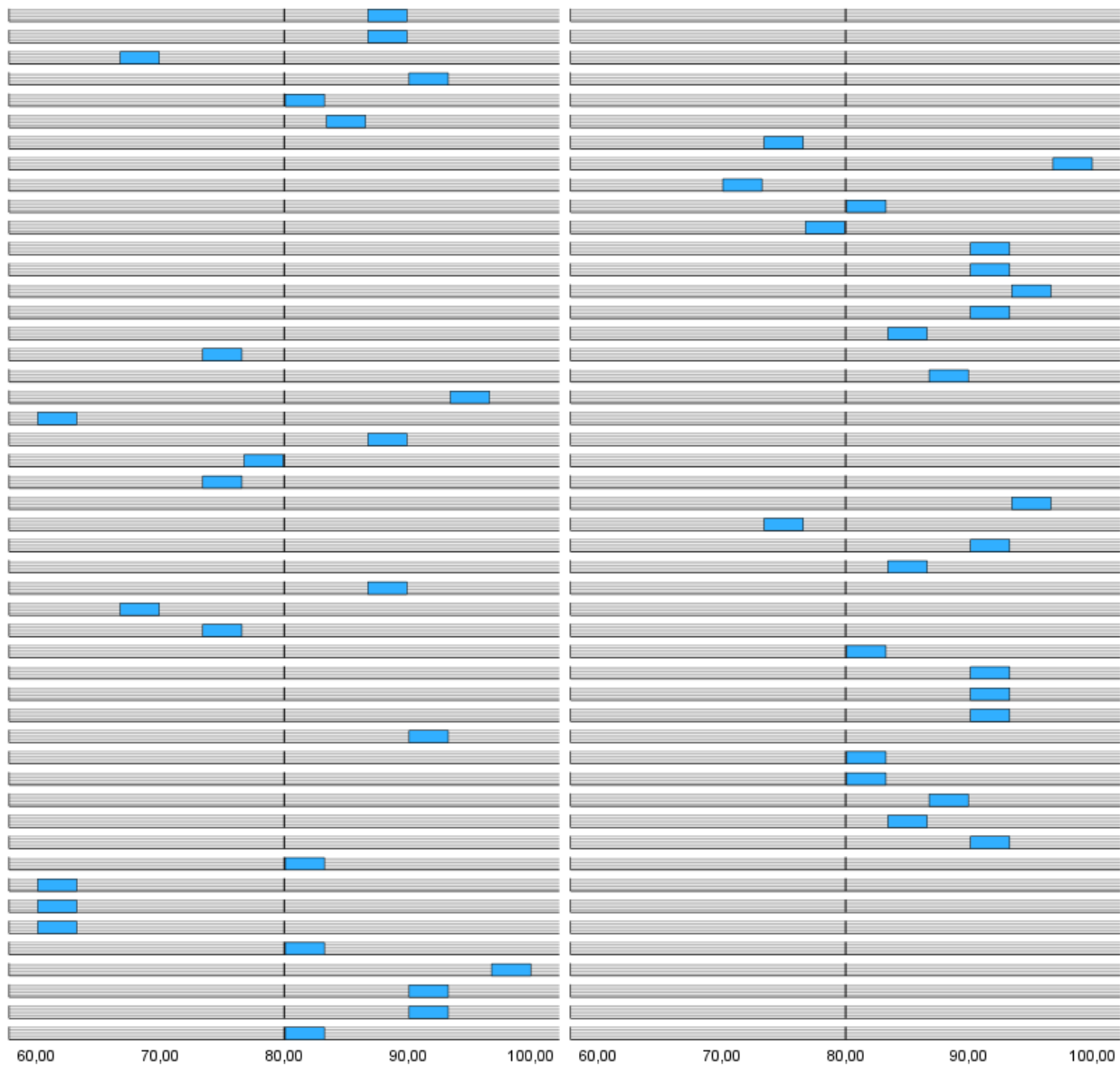

Prosody task (%correct)

TLE

controls

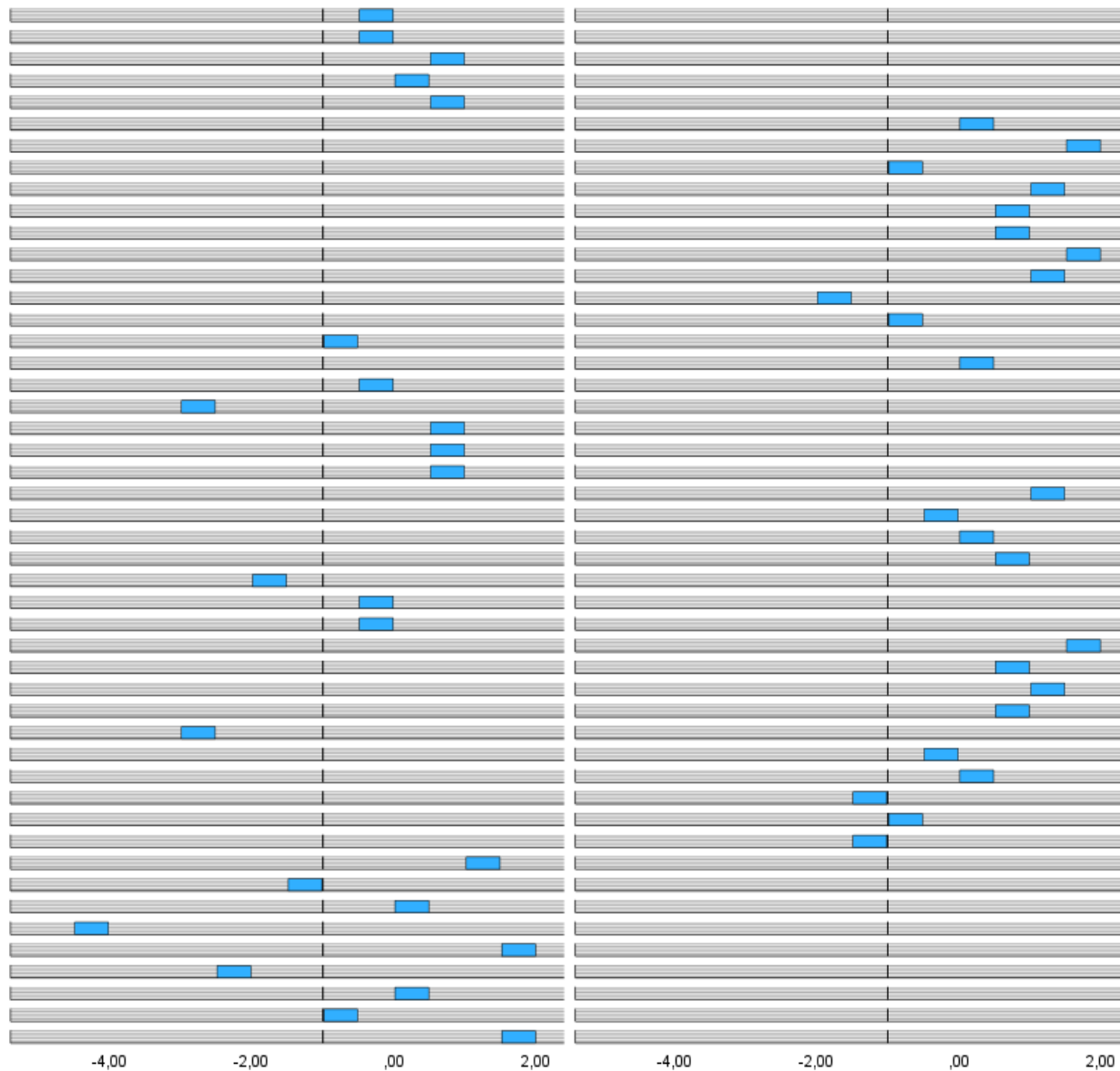

Morph task (BIS)

Supplement: Supplementary file 8 — Supplementary Figure S8. [file 41598_2024_53401_MOESM8_ESM.pdf]

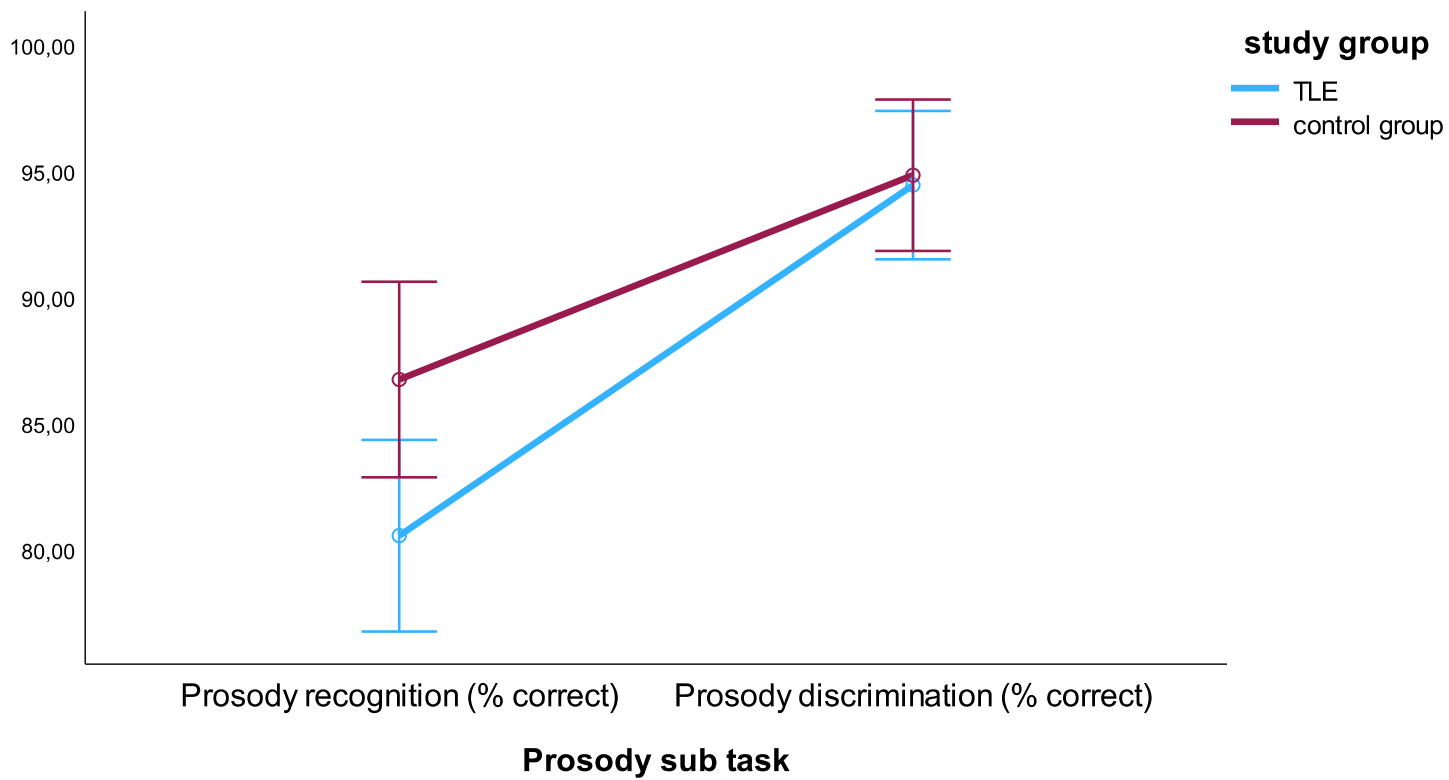

Supplement: Supplementary file 9 — Supplementary Figure S9. [file 41598_2024_53401_MOESM9_ESM.pdf]
