# Supplementary material for: Dynamic facial emotion recognition and affective prosody recognition are associated in patients with temporal lobe epilepsy
Source: Sci Rep. 2024 Feb 16;14:3935. doi: 10.1038/s41598-024-53401-9 (PMC10873350; doi:10.1038/s41598-024-53401-9)
Supplement: Supplementary file 7 — Supplementary Figure S7. [file 41598_2024_53401_MOESM7_ESM.pdf]

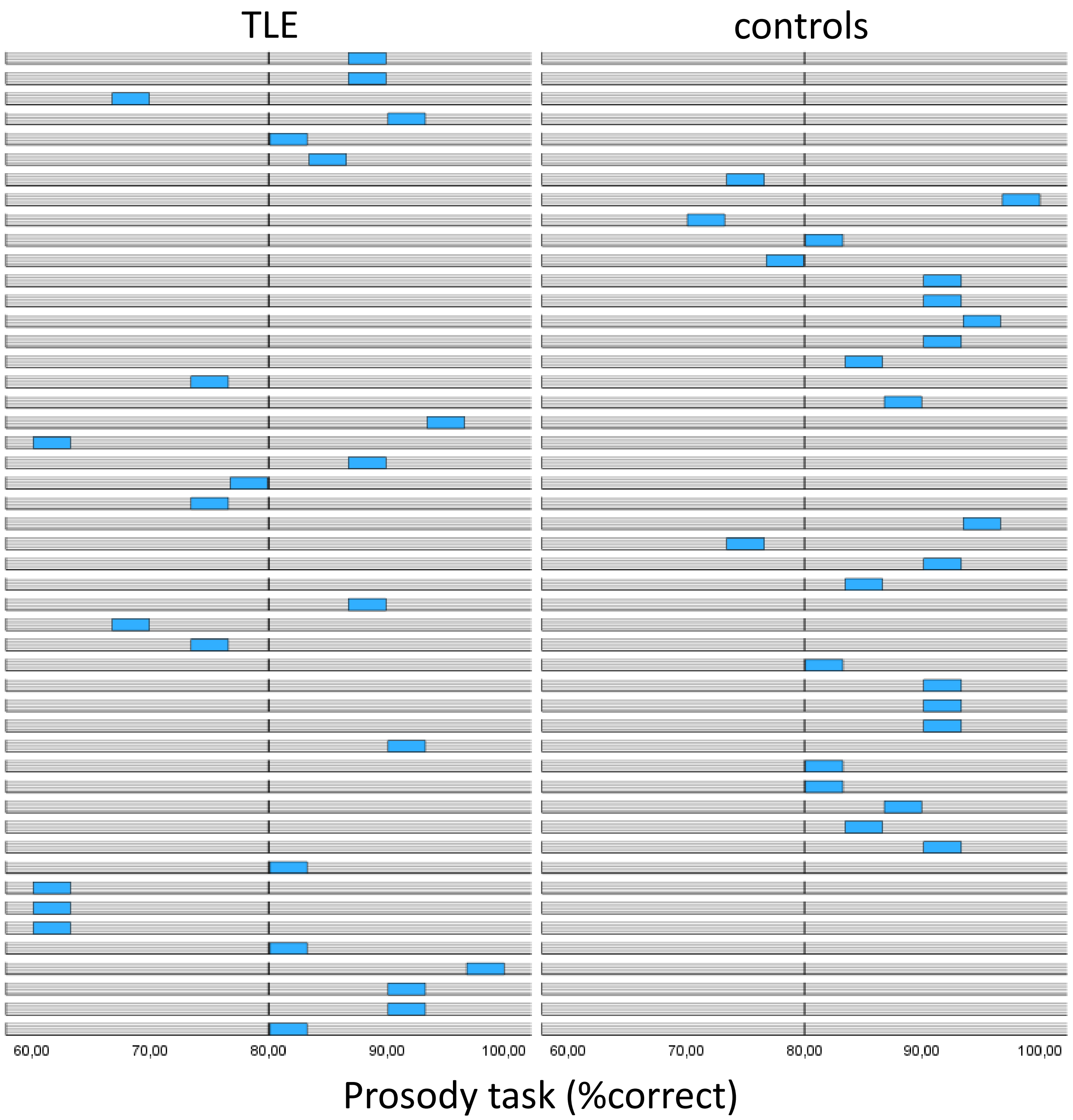

TLE
controls
60,00
70,00
80,00
90,00
100,00
60,00
70,00
80,00
90,00
100,00
Prosody task (%correct)
